# Microbial Steroid Production Technologies: Current Trends and Prospects

**DOI:** 10.3390/microorganisms10010053

**Published:** 2021-12-28

**Authors:** Marina Donova

**Affiliations:** G.K. Skryabin Institute of Biochemistry and Physiology of Microorganisms, Federal Research Center “Pushchino Center for Biological Research of the Russian Academy of Sciences”, Pr. Nauki 5, 142290 Pushchino, Russia; mv_donova@rambler.ru; Tel.: +7-916-541-69-05

This Special Issue aims to collect articles and reviews on new methodologies, research, and achievements in the field of steroid microbial biotechnologies. In recent years, the production of valuable steroids using selected wild-type and engineered microorganisms has become one of the most effective and fast-growing fields of biotechnology and industrial microbiology. There is increasing evidence of growing biotechnology applications of steroid-transforming microorganisms in the pharmaceutical industry, biomedicine, as well as environmental protection from endocrine-disrupting compounds.

Major areas of research include the discovery and engineering of microorganisms capable of producing value-added products from phytosterol and other renewable raw materials, whole-cell, and enzyme biocatalysts performing different reactions of steroid modifications with special attention to the oxyfunctionalization of inactive carbons in steroid molecules and rare steroid production.

Generation of microbial strains with improved biocatalytic features using genetic and metabolic engineering, as well as synthetic biology approaches, cascade bioconversions, new steroid bioproduction schemes, new insights on steroid bioproduction (upstream and downstream processing), the development of new approaches for steroid bioconversion enhancement, and method development for environmental protection from endocrine disruptors are of great importance for the development of steroid biotechnologies.

Steroids are a broad class of terpenoid lipids that typically a contain gonane core of the four fused rings (A–D) composed of 17 carbon atoms and vary mainly by the presence, type and position of functional groups and the side chain at C17. Being essential molecules in all living organisms, steroids play vital functions in vertebrates acting as signal molecules that regulate signal transduction pathways by the binding to the respective intracellular receptors. Another principal function of steroids (such as cholesterol, sitosterol, or ergosterol) is regulating cell membrane fluidity and proper operating of the lipid bilayer. It is believed that steroids originated hundreds of millions of years ago, and in the course of evolution, microorganisms acquired the ability to completely degrade or detoxify steroids through their structural modification, i.e., carry out the biotransformation of steroids [1].

Steroids are ubiquitous in nature and serve as carbon and energy sources for many bacteria. Recent studies showed global distribution of the aerobic microbial degradation pathway in wastewater treatment plants, soil, plant rhizosphere, and the marine environment [2]. Various representatives of Actinobacteria and Proteobacteria are capable of degrading different natural plant and the vertebrate steroids excreted into the environment mainly via the 9(10)-*seco*-pathway [1,3]. The wastes of synthetic hormone production mills may contain residual amounts of steroids that can also be partially oxidized or completely degraded by microorganisms.

Microbiological transformation of plant sterols is currently the technological basis for the production of so-called synthons, from which various pharmaceutical steroids are produced by chemical or combined chemical-enzymatic syntheses. The steroids that can be produced from the synthons are the adrenal cortex hormones, sex hormones, progestins, mineralocorticoids and the non-hormonal steroids for the use in various sectors, including medicine, veterinary medicine, aquaculture, agriculture, food industry. The global needs of the pharmaceutical industry alone for steroid substances exceed 1500 tons annually, and the market for steroid drugs produced from them in 2015 exceeded USD 100 billion, second only to antibiotics. Biotechnologies of microbiological transformation of phytosterols into key synthons of androstane series, such as androstenedione (AD), androstadienedione (ADD), 9-hydroxyandrostenedione (9-OH-AD) have already been implemented on an industrial scale in several countries (USA, Germany, China, India and others). The discovery of new promising steroid molecules with rare bioactivity, including those with antiviral and neuroprotective effects, the growth of the vitamin D market, re-direction of the production schemes to the non-animal raw materials, allows us to predict a significant increase in the demand of the global market for steroid synthons produced by microbiological methods from phytosterols.

Phytosterols are the mixtures of plant sterols (β-sitosterol, stigmasterol, campesterol, brassicasterol and similar) that typically contain 3β-ol-5-en-moiety in the ring A and differ by the aliphatic side chain structure at C17. The composition and quality of phytosterols depends on the production and purification technology applied, but mainly on the source of their industrial production (the wastes of the pulp and paper mills, such as tall oil, or tall pitch; soya beans, the deodorizates obtained in the production of edible vegetable oils and others). Paradoxically, no systematic study of the influence of the composition and quality of phytosterols from different sources on the efficiency of their microbiological transformation has been so far reported.

The achievements in genetic and metabolic engineering have opened a new era in steroid biotechnology. Biotechnology-relevant microbial strains with improved biocatalytic capabilities are being created which are capable of selectively performing various reactions of steroid conversion. Their application may allow replacing multistage and ecologically risky chemical syntheses through environmentally friendly biotechnologies. However, the insufficient selectivity of some bioconversions, low productivity level of the strains in combination with poor aquatic solubility of steroid substrates, toxicity of some steroids for microorganisms are still challenges to effective industrial applications.

To solve the problems, deep understanding of the molecular mechanisms of catabolism of natural sterols and other steroid modifications is necessary. To date, full genome sequencing, assembly of complete genomes and bioinformatic annotation of the gene clusters related to sterol catabolism was performed for many biotechnologically relevant actinobacteria such as *Mycolicibacterium smegmatis* (syn. *Mycobacterium smegmatis*), *Mycolicibacterium neoaurum*, *Mycolicibacterium fortuitum, Rhodococcus jostii*, *Nocardioides simplex* and others. The genomic studies, transcriptomic profiling, proteome and lipidome research, bioinformatic analysis allow identification of the specific genes whose products putatively involved in various reactions of the steroid catabolic pathways. The work on the identification of steroid catabolism genes in biotechnologically relevant actinobacteria, their operon organization, the elucidation of gene functions and their transcription regulation is intensively carried out in many laboratories and research centers in different countries [4,5,6,7]. The main pathways of sterol catabolism are best studied for the biotechnologically relevant strains of *Mycolicibacterium* and *Rhodococcus* genera [8,9,10], however, significant gaps remain in the knowledge on the molecular mechanisms of sterol catabolism related to the function of a number of genes and regulation of their transcription.

The progress made in recent years in understanding the pathways of microbial steroid catabolism and the development of genetic tools for manipulating with actinobacteria has led to the creation of effective genetically engineered strains that produce C_19_-steroids, -androstenedione (AD), androstadienedione (ADD) and 9α-hydroxyandrostenedione (9-OH-AD), from phytosterol. For example, inactivation of three genes coding for 3-ketosteroid-∆^1^-dehydrogenases (KstD) and augmentation of the genes encoding reductase component of 9α-hydroxylase in *M. neoaurum* ATCC 25795 provided production of 9-OH-AD from phytosterol [11]. Increase in the number of copies of the gene *cyp125* related to the initial stages of sterol side chain oxidation, and of the genes related to rings A/B oxidation (*choD*, *3-hsd*) in a mutant strain of *M. neoaurum* ∆*kstD*, ∆*kshB* (i.e., with deletions in the genes whose products are responsible for steroid core destruction) improved the strain productivity and selectivity of AD production from phytosterol [12,13]. Attempts are being made to increase the productivity of strains by changing the permeability of the cell wall, for example, by reducing the synthesis of lipoarabinomannan (inactivation of the *embC* gene) in a mycobacterial AD producer [14], as well as stimulating central metabolism [12]. These are just a few examples that demonstrate the close interest of researchers in the improving the properties of mycobacterial producers of the key synthons, -AD, ADD, 9-OH-AD.

Microbial production of steroids with a partially oxidized side chain (C_22_-, C_24_-steroids) from phytosterol is less studied. The significant achievement in this area is the creation of a recombinant strain of *M. neoaurum* with deletions in four genes related to steroid core destruction and the last round of side chain oxidation and the increase in the copy number of four other genes encoding the initial stages of the side chain oxidation. This made it possible to switch the metabolic routes towards the predominant formation of 23,24-bisnorholenic acid (BNC), a close precursor in progesterone synthesis [15].

These few examples, of course, do not reflect the whole picture, but illustrate that current research in the field of microbiological conversion of natural sterols is mainly focused on modifying the known microbial producers (such as *M. neoaurum*) to improve their productivity and selectivity of sterol bioconversion to the main synthons (AD, ADD, 9-OH-AD), or switch the metabolic pathways to the production of C_22_-steroids (BNC) from phytosterol. Numerous publications are devoted to the development of biotechnological processes for the production of these synthons from phytosterol based on the obtained mutant or recombinant strains (for review, see [12]).

Much less information is available on the so-called “mycolate-free” steroid-transforming actinobacteria such as representatives of *Pseudonocardia*, *Nocardioides*, *Saccharopolyspora* and others. Meanwhile, these actinobacteria certainly possess biotechnological potential, for example, for the development of bio-processes for the production of active forms of vitamin D (25-hydroxylation) [16], as well as new antiviral drugs based on hydroxy derivatives of sterols [17], or production of other bioactive steroids [18]. The search and study of the catabolic features of new microbial strains for possible biotechnological applications have not lost their relevance.

An important task is to expand the set of synthons produced from phytosterol in a single biotechnological step, which will significantly simplify technological schemes and replace environmentally risky and multi-stage chemical syntheses. The examples of essential synthons and the pharmaceutical steroids that can be produced from them are given in Figure 1. The creation of recombinant strains capable of the production of oxyfunctionalized steroids is of great importance.

Along with microbiological transformation of sterols, oxyfunctionalization of steroids is an area where chemical methods are either very difficult, or even impossible to implement. As known, the presence of oxygen (mainly, hydroxyl) functions in the steroid molecule provides increase in the polarity of hydrophobic steroid molecules, the decrease in their toxicity, better transport through cell membranes and, in general, improves biological effects of steroids. The regio- and stereo-position, and the number of introduced hydroxyl groups are of importance. For example, the presence of a hydroxyl group at position 11β is important for anti-inflammatory activity (for example, hydrocortisone, prednisolone), 16α-hydroxyl plays a role in the bioactivity of glucocorticoids (triamcinolone, dexamethasone, etc.), cardioactive steroids have a 14β-hydroxyl group [20], the presence of hydroxyls at C7 in derivatives of dehydroepiandrosterone (DHEA) and epiandrosterone (EpiA) affects the neuroprotective activity [21]; active forms of vitamin D3 contain 1α- and 25α-hydroxyl groups [22]; the presence of 7α- and 7β-hydroxyl functions plays an important role in the bioactivity of many steroids [23].

Single-stage production of target hydroxysteroids from natural sterols is possible by heterologous expression of cytochrome-P450 monooxygenase systems in mycobacterial hosts. For example, heterologous expression of 11α-hydroxylase system from the fungus *Rhizopus oryzae* in *M. smegmatis* allows single stage production of 11α-hydroxyandrostenedione from phytosterol [24].

As known, unlike bacteria, filamentous fungi do not utilize steroids as carbon and energy sources, but mainly detoxify them as fungitoxic molecules by introduction of hydroxyl groups into various positions of the steroid substrate. Some biotechnologies of hydroxylation based on filamentous fungi have been implemented at the industrial level. For example, industrial production of hydrocortisone is based on chemical synthesis with an essential stage—11β-hydroxylation carried out by the fungus *Curvularia lunata* (*Cochliobolus lunatus*) at a scale of 100 tons annually [25]. However, practical implementation of the hydroxylation bioprocess based on the wild-type fungal strains is often limited due to the problems associated with the presence of several different hydroxylase activities in the fungal strains, low specificity of cytochrome P450 monooxygenases which accounted for steroid hydroxylation to form the mixture di-, tri-, and polyhydroxylated steroids, or a mixture of the stereoisomers. In addition, the presence of side activities in wild-type fungal strains also complicates the purification procedures of target hydroxylated products.

The solution can be heterologous expression of fungal hydroxylase systems in bacterial and yeast hosts. Current research in this area is focused on creating genetically engineered strains with hydroxylating activity [26,27,28,29,30,31]. Multiple reports concern the expression of 11α-hydroxylase systems of fungi (*Aspergillus ochraceus*, *R. oryzae*, *Absidia coerulea*) in the yeast hosts (*Pichia pastoris*, *Saccharomyces cerevisiae*, *Shizosaccharomyces pombe*). An undoubted achievement is the creation of *Corynebacterium glutamicum* recombinant strain producing hydrocortisone by expressing the 11β-hydroxylase system from of the fungus *C. lunatus* [29]. Although the low productivity of the engineered microorganisms is low, it can be predicted that the development of this direction may lead to significant practical achievements in the near future.

Study of microbial diversity and metabolic capabilities of steroid transforming microorganisms, discovery of new microbial activities, creation of novel industrial microorganisms capable of effectively producing valuable steroids from renewable natural sources, development of the cost-effective and ecofriendly biotechnologies to replace multistage and ecologically risky chemical syntheses are the basic trends in the field. Novel findings in these areas will contribute, not only to the pharmaceutical industry oriented to the higher life quality, healthy development and ageing, but also to the economy branches where the steroids are applied.

## Figures and Tables

**Figure 1:**
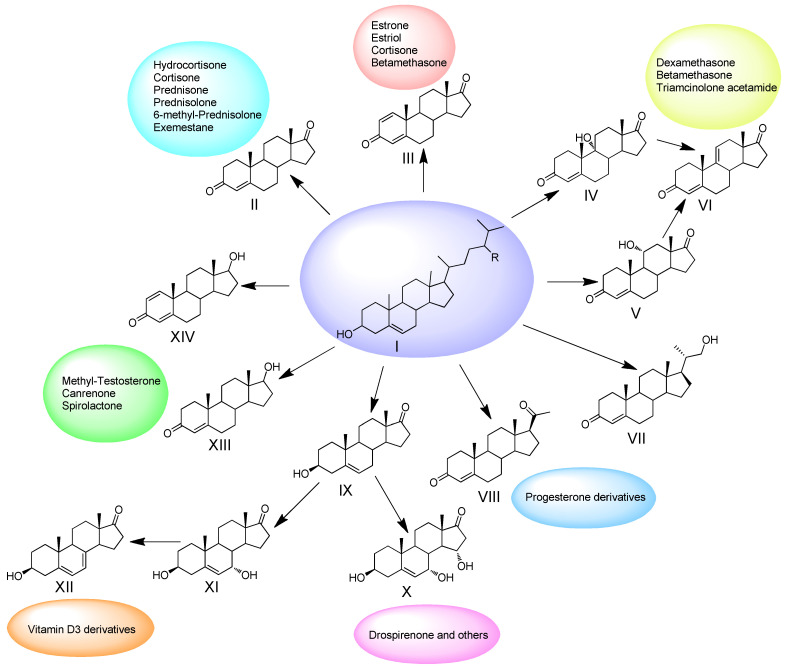
Examples of the steroid synthons produced from phytosterol (I) through microbiological transformations and the pharmaceutical steroids that can be synthesized on their base: II—androst-4-ene-3,17-dione (AD); III—androst1,4-diene-3,17-dione (ADD); IV—9α-hydroxyandrost-4-ene-3,17-dione (9-OH-AD); V—11α-hydroxyandrost-4-ene-3,17-dione; VI—androst-4,9(11)-diene-3,17-dione; VII—20-hydroxymethylpregn-4-ene-3-one (20-HMP, BA); VIII—progesterone; IX—3β-hydroxyandrost-5-en-17-one (dehydroepiandrosterone; prasterone, DHEA); X—3β,7α,15α-trihydroxyandrost-5-ene-17-one; XII—3β-hydroxy-5,7-diene-17-one; XIII—androst-4-ene-3-one-17β-ol (testosterone); XIV—androst-1,4-diene-3-one-17β-ol, boldenone (in accordance with [19]).

## Data Availability

Not Applicable.
